# The complete chloroplast genome of *Cymbidium serratum* (Orchidaceae): a rare and Endangered species endemic to Southwest China

**DOI:** 10.1080/23802359.2020.1775514

**Published:** 2020-06-11

**Authors:** Lei Shao, Huijuan Ning

**Affiliations:** Zhejiang Provincial Key Laboratory of Germplasm Innovation and Utilization for Garden Plants, Key Laboratory of National Forestry and Grassland Administration on Germplasm Innovation and Utilization for Southern Garden Plants, School of Landscape Architecture, Zhejiang Agriculture and Forestry University, Hangzhou, China

**Keywords:** *Cymbidium serratum*, endangered species, chloroplast genome, phylogenetic analysis

## Abstract

*Cymbidium serratum* is a dominant species in the large orchid family with beautiful flowers, thick petals, and long flowering periods, and has a long history of cultivation in Southwest China. However, its wild resources have been threatened with extinction due to environmental degradation and artificial exploitation. In this study, the complete chloroplast genome of *C. serratum* was obtained through Illumina sequencing. The size of chloroplast genome of *C. serratum* is 149,998 bp, including large single-copy (LSC) and small single-copy (SSC) regions over 84,854 bp and 13,926 bp, respectively, and two inverted repeats (IRs) of 25,609 bp. The total GC content was 37.11%. The chloroplast genome contains 129 genes, including 83 protein-coding genes, 8 rRNA genes, and 38 tRNA genes. The maximum-likelihood phylogenetic tree indicated that *C. serratum* is a sister species with the clade composed of *C. faberi* and *C. goeringii*. The complete chloroplast genome of *C. serratum* will contribute to protecting this highly endangered species, and provide genetic information about genetic diversity and sustainable use.

*Cymbidium serratum* (Orchidaceae) is one of the eight traditional Chinese orchids. It is mainly distributed in the Yunnan Plateau, also in the mountainous areas with high altitudes at the junction of Sichuan, Guizhou, and Yunnan (Zhaoyang et al. 2010; Dagui 2011). *Cymbidium serratum* has extremely colorful flowers, which can be attributed to factors such as complex terrain, diverse climate types, strong ultraviolet rays in high altitude areas (Yufang 2005; Weiqing 2014). *Cymbidium serratum* is very limited in resources because of its narrow growing area (Yufang 2005). In recent years, due to the deterioration of the ecological environment and repeated exploitation, the wild resources of *C. serratum* have been Endangered (Huixian et al. 2010). In view of its threatened situation, it is urgent to strengthen the resource conservation and genetic breeding research of the species. Since the chloroplast genome sequence is inherited from matrilineal plants in most angiosperms and contains a great quantity of valuable genetic information (Daniell et al. 2016; Gitzendanner et al. 2018), it is widely used in species conservation, genome evolution and phylogenetic studies at various species levels (Barrett et al. 2013; Turmel et al. 2019). In this study, the complete chloroplast genome of *C. serratum* is reported providing valuable genetic information for further research on genetic diversity and conservation of this highly Endangered species.

The fresh leaves of *C. serratum* were collected from Bijie (105°37′59.16″E; 27°29′14.98″N), Guizhou Province, China. Voucher specimen was deposited at Orchid Resource Nursery of Zhejiang Agriculture and Forestry University (specimen code ZAFU20140310). The total genomic DNA was extracted by modified CTAB method (Fu et al. 2017) and sequenced by NovaSeq platform (Illumina, USA). The complete cp genome was assembled using SPAdes v3.10.1 (Bankevich et al. 2012) and genome annotation was performed with Geneious ver. 10.1 (Kearse et al. 2012). The whole chloroplast genome sequence of *C. serratum* has been submitted to GenBank (accession number MT273089).

The size of chloroplast genome of *C. serratum* is 149998 bp, including large single-copy (LSC) and small single-copy (SSC) regions over 84,854 and 13,926 bp, respectively, and two inverted repeats (IRs) of 25,609 bp. The total GC content is 37.11%, and the GC content of LSC, SSC, and IR are 34.46, 29.63, and 43.53%, respectively. The chloroplast genome of *C. serratum* contains 129 genes, including 83 protein-coding genes, 8 rRNA genes, and 38 tRNA genes. There are 19 genes duplicated in the IRs, and the remaining 110 genes are unique genes. Thirteen genes contain one intron, while three genes have two introns.

To confirm the phylogenetic status of *C. serratum*, phylogenetic analysis was performed based on the complete chloroplast genomes of nine *Cymbidium* species and two Orchidaceae species as outgroups downloaded from GenBank. MAFFT v7.388 (Nakamura et al. 2018) was applied for multisequence alignment, and the aligned sequences were employed to construct the maximum-likelihood phylogenetic tree using RAxML v8.2.10 (Stamatakis 2014) with 1000 bootstrap. The ML phylogenetic analysis manifests that *C. serratum* is a sister species with the clade composed of *C. faberi* and *C. goeringii* (Figure 1).

**Figure 1.**
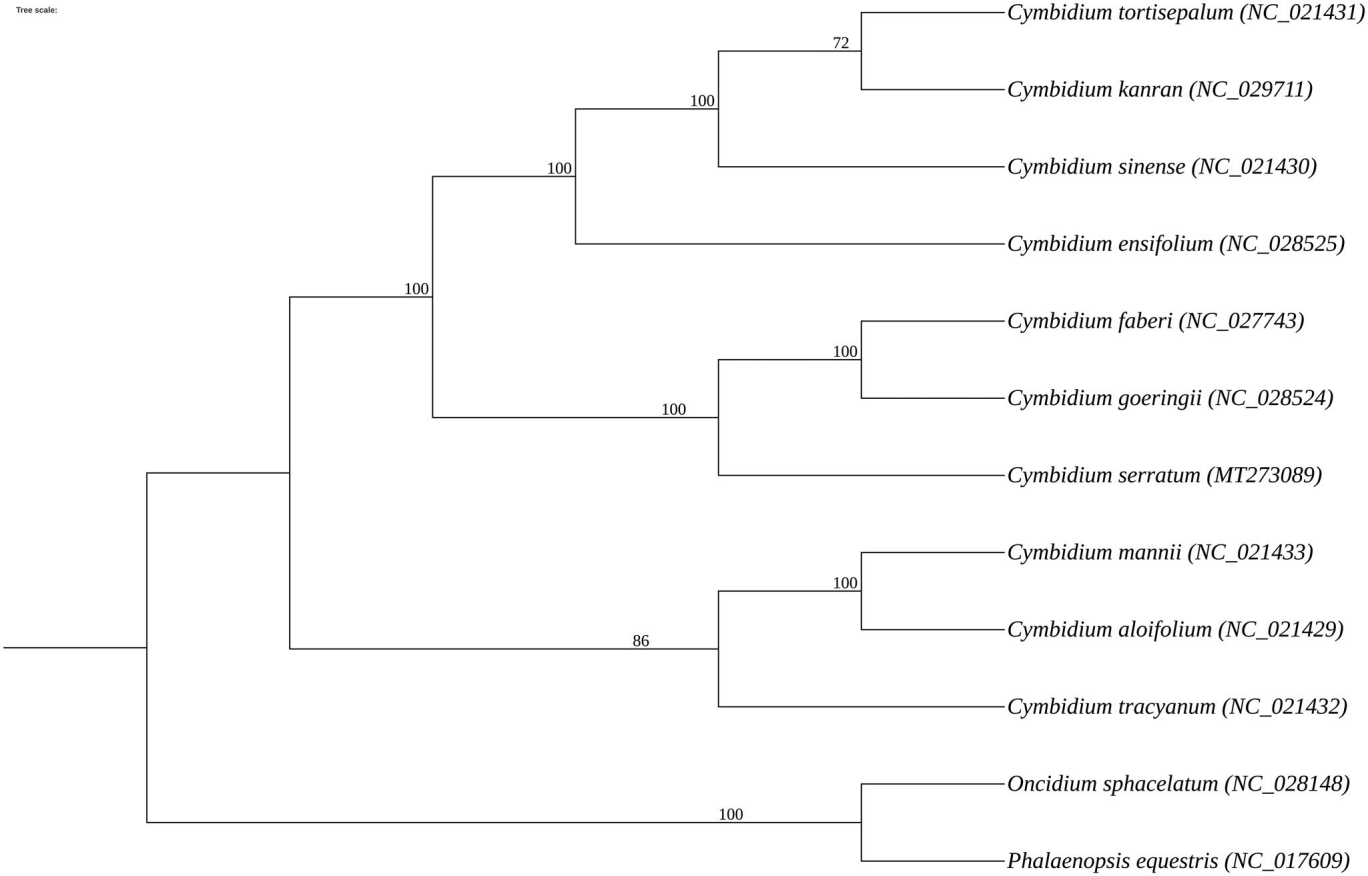
Maximum-likelihood phylogenetic tree based on 12 complete chloroplast genomes of Orchidaceae. Numbers in the nodes indicate the bootstrap support values from 1000 replicates.

## Data Availability

The data that support the findings of this study are openly available in GenBank at https://www.ncbi.nlm.nih.gov, GenBank Accession Number: MT273089.
